# The Consumption of Sweets and Academic Performance among Mongolian Children

**DOI:** 10.3390/ijerph17238912

**Published:** 2020-11-30

**Authors:** Noboru Nakahara, Yusuke Matsuyama, Shiho Kino, Nomin Badrakhkhuu, Takuya Ogawa, Keiji Moriyama, Takeo Fujiwara, Ichiro Kawachi

**Affiliations:** 1 Department of Global Health Promotion, Tokyo Medical and Dental University, Bunkyo-ku, Tokyo 113-8519, Japan; 160641ms@tmd.ac.jp (N.N.); matsuyama-thk@umin.org (Y.M.); 2 Department of Social and Behavioral Science, Harvard T.H. Chan School of Public Health, Boston, MA 02115, USA; shiho.kino.lucky@gmail.com (S.K.); ikawachi@hsph.harvard.edu (I.K.); 3 Department of Maxillofacial Orthognathics, Graduate School of Medical and Dental Sciences, Tokyo Medical and Dental University, Tokyo 113-8510, Japan; nominchik@mail.ru (N.B.); t-ogawa.mort@tmd.ac.jp (T.O.); k-moriyama.mort@tmd.ac.jp (K.M.)

**Keywords:** academic score, nutrition, Mongolia

## Abstract

The regular consumption of sweets has been shown to have an adverse association with the academic performance of children in developed countries; however, the situation in developing countries is less clear. Therefore, we examined the association between the consumption of sweets and academic performance among Mongolian children via a cross-sectional study employing data from 787 children aged 8–16 from two public schools in Ulaanbaatar, the capital of Mongolia. The frequency of the consumption of sweets by the children was captured using a questionnaire and then linked to their academic scores; the association between the consumption of sweets and scores in mathematics and the Mongolian language was evaluated using multiple linear regression adjusted for other covariates. It was found that out of 787 students, 58.6% ate sweets every day. After adjusting for covariates, no significant association was observed between the consumption of sweets and mathematics scores (coefficient: 0.15; 95% confidence interval (CI): −0.02–0.32), while a higher consumption of sweets was significantly associated with higher scores in the Mongolian language (coefficient: 0.25; 95% CI: 0.09–0.41). The associations established in this study are inconsistent with the reports of other studies.

## 1. Introduction

Previous studies have reported that a lower intake of energy-dense, nutrient-poor foods (such as sweets and candy) is associated with better academic performance [1]. Many studies on this topic have been conducted in developed countries including the US [2,3,4,5,6,7], Iceland [8,9], the UK [10], Norway [11] and Korea [12]. The study conducted in the UK, for example, reported a negative correlation between junk food consumption prior to school entry and academic achievements; this was explained by the negative effect of low nutrition quality upon cognitive outcomes [9]. Similar results were found among American children and this was explained by the effect of a high fat diet upon cognitive function [2].

The population segments consuming unhealthy foods differ between developed and developing countries. According to the nutrition transition theory, economic development and urbanization are associated with distinct changes in nutrition patterns among different population segments [13]. As a by-product of economic growth and integration, westernized dietary patterns (the consumption of processed foods, high sugar/high fat foods, sugar-sweetened beverages and fast food) have spread across the globe including to developing countries [14]. Initially, those with higher incomes living in urban areas start consuming westernized foods [13]; this trend continues until the knowledge of nutrition spreads across higher socioeconomic status (SES) groups who then begin to adopt a more prudent dietary pattern [13]. At this point, the consumption of highly processed food begins to shift downward to lower SES population segments [13].

Given these differing patterns of unhealthy food consumption, findings from developed countries may not be directly applicable to developing countries. More specifically, in developed countries, people from lower SES backgrounds are more likely to consume unhealthy foods such as sugar-sweetened beverages [15]; thus, SES can be a confounder between food consumption and academic performance. While the aforementioned previous studies [2,3,4,5,6,7,8,9,10] have adjusted their results for SES-related factors (e.g., parental income and education), such statistical adjustments do not necessarily guarantee their ability to extrapolate their findings to developing countries. On the other hand, in developing countries people with a higher SES are more likely to consume calorie-dense foods [16] than in developed countries such as the US [17]; however, few studies have evaluated the association in developing countries [18].

Mongolia is among the developing countries in the middle of the nutrition transition with children in urban areas consuming more sweets than those in rural areas [19]. It would be informative to investigate the relationship between the consumption of sweets and academic performance in such a country. This study aimed to investigate the association between the consumption of sweets and the academic performance of children in Mongolia.

## 2. Materials and Methods

### 2.1. Study Population

This study was conducted using cross-sectional data from a longitudinal survey of schoolchildren in the capital of Mongolia, Ulaanbaatar. The original purpose of the survey was to collect maxillofacial morphological data from the general population of Mongolian children, inspect the association of these data via socioenvironmental and genetic factors and compare the results with data from Japan to reveal the cause of malocclusion [20,21]. The survey was conducted in two public schools, the biggest in each of the two largest districts in Ulaanbaatar (i.e., Bayanzürkh and Songino Khairkhan). Children in the first and second classes for each grade between four and ten were included in the survey. Further details concerning the survey have been reported elsewhere [20,21]. This study was approved by the Ethical Review Board of the Mongolian National University of Medical Science (No. 13-12/1A) and Tokyo Medical and Dental University (No. D2013-071). Informed consent for this study was obtained from caregivers and children. Consent to linking to the academic performance of participating children was obtained using the opt-out procedure and the collected records were anonymized for analysis.

**Figure 1:**
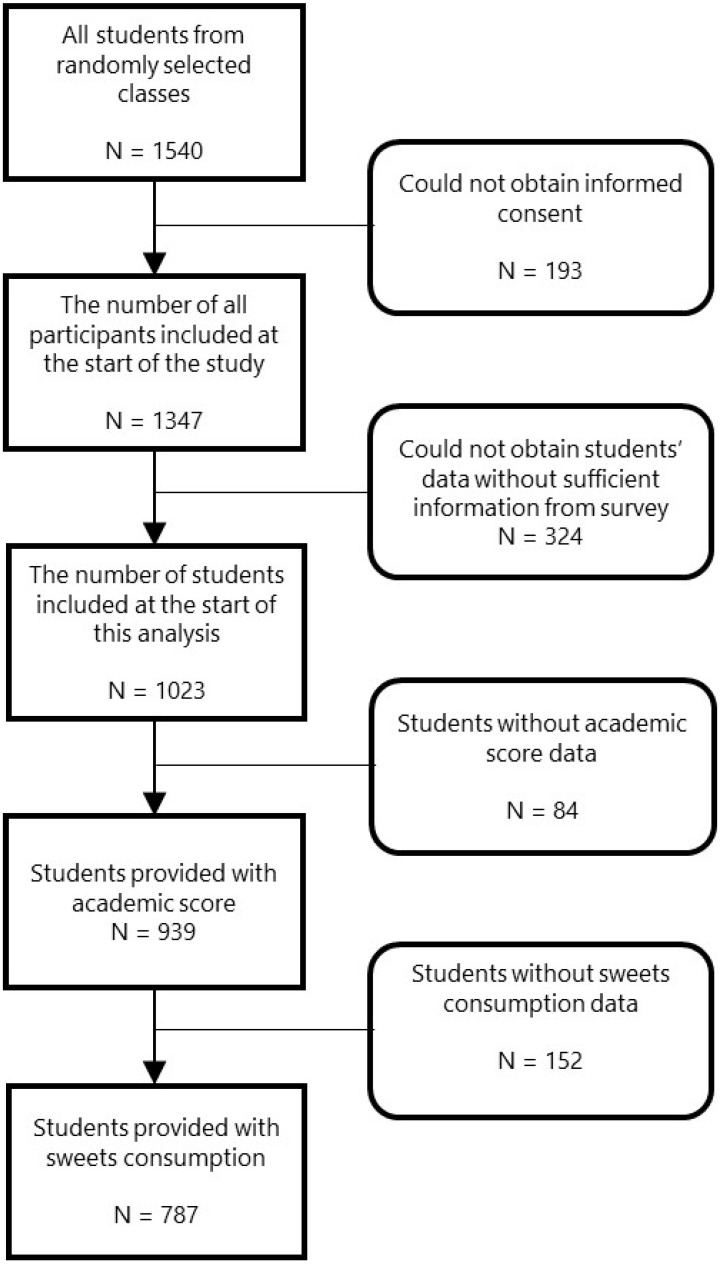
Flowchart for selecting participants in the study.

Figure 1 shows the flowchart used to select participants for the study. Out of the 1540 students from all selected classes, 193 were excluded because we could not obtain informed consent (response rate: 87.5%). Of the remaining 1347 students, 324 were excluded due to the lack of information and omitted from the dataset before this analysis was conducted. In addition, information on academic performance or the consumption of sweets was missing for 84 and 152 participants, respectively, resulting in 787 students being included in the analysis.

### 2.2. Measurements

The final grades for students provided by schools at the end of the school year were used as academic performance data. In Mongolia, all public schools have the same criteria for marking students, which were established by the government; marks are assigned from 0 to 100 with 60 being the passing mark. Among 20 subjects studied by the selected students across various grades, mathematics and the Mongolian language were studied at all grades; thus, they were selected as targets for the present study. For the purpose of this analysis, marks were standardized for each school grade for ease of comparison across different grades.

Students were asked to report on the frequency with which they consumed 25 food items (listed in Appendix A) over the previous month by choosing one of the following options for each item: “never had that food within the past month”, “consumed less than once a week”, “consumed once a week”, “consumed 2 to 3 times a week”, “consumed 4 to 6 times a week”, “consumed once a day”, “consumed twice a day” and “consumed more than three times a day”. Sweets were defined as snacks mainly containing sugar such as candies or chocolates; however, no food examples were provided in the questionnaire. Thus, the types of food considered to be sweets might have differed between children. This variable was dichotomized by the median (eat every day or less than every day) in the presented regression analysis.

Several other factors relevant to both academic performance and dietary habits were also evaluated by the questionnaire. Parents were asked to choose among the following six categories to indicate their household average monthly income: ≤200,000 tögrögs, 210,000–500,000 tögrögs, 510,000–800,000 tögrögs, 810,000–1,000,000 tögrögs, 1,000,000–1.8 million tögrögs and >1,800,000 million tögrögs (100,000 tögrögs ≈ 34 USD at the time of writing). For the purpose of this analysis, these categories were merged into three: <500,000 tögrögs, 500,000–1,000,000 tögrögs and >1,000,000 tögrögs. The education level of mothers was reported to be in one of the following five categories: never received a formal education, elementary or junior high school, high school graduate, vocational college and college graduate or higher. This variable was merged into three categories: primary school, secondary school to vocational college or college graduate and higher. The regularity with which students ate breakfast was assessed according to the following categories: never, sometimes and every day. Physical activity, apart from physical education classes at school, was categorized as occurring never, once a week, two to three times a week, four to six times a week and every day. Student mental health was evaluated using the strengths and difficulties questionnaire (SDQ) [22], which was used in a previous study to screen emotional and behavioral problems among Mongolian adolescents [23]. The SDQ score was categorized into the following bands: ≤15 (close to average), 16–19 (slightly high) and ≥20 (high). The body mass index (BMI) of the students was calculated from their heights and weights measured in schools with clothes on but without shoes. The BMI was used as a continuous variable in this analysis.

### 2.3. Statistical Analysis

The association between the consumption of sweets and academic performance was evaluated using multiple linear regression analysis. To consider potential confounders, the following three models were constructed through the sequential addition of covariates: a crude model (Model 1); a model adjusted for gender, BMI, household income, mother’s education, regularity of eating breakfast, SDQ score and physical activities (Model 2) and a model further adjusted for dietary habits reported to be associated with academic performance such as the consumption of junk food (instant noodles, chips, juice) [1], vegetables [1], fruits [1] and milk [5] (Model 3). Two meat items frequently consumed in Mongolia, mutton and beef, were also adjusted in Model 3. The table in Appendix A shows the correlation between the consumption of sweets and other food items. Missing information concerning covariates was imputed using the mice R 3.6.0 software (The R Foundation for Statistical Computing, Vienna, Australia) package. Ten multiple imputations were performed using the classification and regression trees (CART) method [24]. *P*-values below 0.05 were considered to indicate a statistical significance. All analytical stages except multiple imputation were performed using Stata v.14 (StataCorp LP, College Station, TX, USA).

## 3. Results

### 3.1. Distribution of Participant Characteristics

Table 1 lists the distribution of participant characteristics according to the frequency at which sweets were consumed. A total of 58% of children were found to consume sweets every day. Older age, higher school grade and not eating breakfast regularly were significantly associated with the consumption of sweets every day. Maternal education was positively associated with the consumption of sweets although it was not statistically significant (*p* = 0.057). Household income was not associated with the consumption of sweets (*p* = 0.560) nor were exercise and problematic behaviors (*p* = 0.237).

### 3.2. Association between the Consumption of Sweets and Mathematics Scores

Table 2 shows the association between the consumption of sweets and academic scores in mathematics. According to the crude model (Model 1), children who consumed sweets every day had significantly higher scores in mathematics (coefficient: 0.15; 95% confidence interval (CI): 0.01–0.29); however, this model did not largely explain the variation in the mathematics score (adjusted R squared: 0.01) and the association became insignificant after adjusting for gender, BMI, mother’s education, breakfast, SDQ, exercise (Model 2; coefficient: 0.12; 95% CI: –0.02–0.26) and consumption of other foods (Model 3; coefficient: 0.15; 95% CI: –0.02–0.32). Small portions of the variation in the mathematics score were explained by these models (adjusted R squared: 0.08 and 0.06, respectively); according to Model 3, being male, higher BMI, a higher SDQ score and a habit of consuming vegetables every day was significantly associated with lower scores in mathematics. The variance Inflation Factor (VIF) calculated from Models 2 and 3 did not suggest multicollinearity (all VIFs < 2).

### 3.3. Association between the Consumption of Sweets and Mongolian Language Score

Table 3 shows the association between the consumption of sweets and the Mongolian language score. According to the crude model (Model 1), children consuming sweets every day had significantly higher scores in the Mongolian language (coefficient: 0.25; 95% CI: 0.11–0.39). This model did not largely explain the variation in the Mongolian language score (adjusted R squared: 0.02). The association with language remained significant even after adjusting for gender, BMI, mother’s education, breakfast, SDQ, exercise (Model 2; coefficient: 0.21; 95% CI: 0.08–0.34) and the consumption of other foods (Model 3; coefficient: 0.25; 95% CI: 0.09–0.41). These models partly explained the variation in the Mongolian language score (adjusted R squared: 0.18 and 0.18, respectively). According to Model 3, being male, higher BMI, a lower household income and a higher SDQ score was significantly associated with lower scores in the Mongolian language. The VIF calculated from Model 2 and Model 3 did not suggest multicollinearity (all VIFs < 2).

## 4. Discussion

Consuming sweets every day was shown to be associated with higher academic performance in mathematics and the Mongolian language among Mongolian school children; however, this association was not statistically significant for mathematics after adjusting for covariates. This result is inconsistent with other studies that were mainly conducted in developed countries and implies that such studies may not be applicable to children in the developing world. Other food items were not significantly associated with academic performance.

The positive association between the consumption of sweets and the Mongolian language score might be explained by a previous study suggesting that modest increases in blood glucose improves learning and memory function [25]. A previous experimental study showed that male undergraduates who consumed sugary snacks showed higher performance in memory tasks compared with the control group [26]. At the same time, we found a relationship (though not statistically significant) between a higher household SES (for which maternal education was a proxy) and the daily consumption of sweets. This result made us suspect that the correlation between the consumption of sweets and academic performance was residually confounded by SES. In developed country settings, socioeconomic disadvantage is correlated with both the consumption of sweets and lower academic performance. In Mongolia, a higher SES is associated with a higher consumption of sweets and higher academic performance. If the consumption of sweets was causally related to academic performance, the same association would have been observed (i.e., more sweets lead to lower academic scores) in Mongolia. In addition to the main finding, no association could be found between breakfast consumption and academic performance; this contradicts previous studies reporting that breakfast consumption may improve cognitive function [27]. While not being statistically significant, an association was established between physical activity and better academic performance.

Studies on the association between junk/fast food consumption and academic performance have mainly been conducted in developed countries; many of these studies suggest that these two metrics are negatively correlated [7,8,9,11]. According to the nutrition transition theory, SES can be seen as a confounder of unhealthy food choices and low academic performance in developed countries because highly processed and energy-dense foods are cheaper and more affordable (cost per calorie) than fresh produce [28].

By contrast, the correlation between socioeconomic disadvantage and unhealthy foods has not (yet) emerged in less developed countries such as Mongolia, where transnational junk food manufacturers have a low market penetration [29]. If there was a true causal relationship between the consumption of sweets and academic performance, the association ought to be replicated even in developing countries during the early stage of the nutrition transition; however, our study observed a positive correlation between the consumption of sweets and academic performance. There was also a positive association between the consumption of sweets and urban residence in the National Nutrition Survey conducted by the National Center for Public Health of Mongolia in 2017, which focused upon the prevalence of nutrition conditions [19].

Students’ SDQ scores were found to have a significant inverse association with their school scores whereas their SES was positively associated with school scores. These findings are consistent with those of previous studies; for example, a self-reported SDQ score was negatively associated with academic test performance in a Danish national birth cohort [30]. Many studies have reported that socioeconomic context is the key to academic success [31,32,33]; by contrast, SDQ scores were not significantly associated with the consumption of sweets in this study. Despite some early findings, a solid causal relationship between the consumption of sweets and problematic behavior or hyperactivity has yet to be established [34].

## 5. Limitations and Recommendations for Future Research

First, while the data on the students’ academic performance were validated by the schools, food consumption data were self-reported. We divided the consumption of sweets variable by its median (eat every day or less than every day) to obtain a sufficient number of respondents in each group. Thus, we were unable to assess the dose-response relationship. Second, causal inferences cannot be drawn because this was a cross-sectional study with a small sample size. As we excluded children for whom information on academic performance and the consumption of sweets was lacking, the results might be biased. However, it was difficult to speculate upon whether the results were over- or underestimated because, of the 236 children excluded from the analysis, 218 lacked information on the consumption of sweets. Third, our results may also not be generalizable to rural areas in Mongolia or other developing countries. The participants were not asked about their place of residence, meaning that their SES could not be assessed precisely. Finally, only mathematics and the Mongolian language were considered in this study; academic scores in other subjects and the children’s non-cognitive skills are yet to be studied. Further research is needed to investigate the different associations found for mathematics and Mongolian language scores.

## 6. Conclusions

The present study suggests that previous studies linking the consumption of sweets and academic performance may have been weakly confounded by SES. Our data were collected from Mongolia, a country in the midst of the nutritional transition to a more developed world pattern. The consumption of sweets was positively associated with academic scores in Mongolian language classes but the association with mathematics scores was not statistically significant. Thus, the findings of the present study must be carefully interpreted. Accurate measurement of household SES should help further understand the association between food intake patterns and academic performance.

## Figures and Tables

**Table 1:** Study population characteristics (*n* = 787).

	The Consumption of Sweets				
	Eat Every Day		Less Than Every Day		
	*n* or Mean	% or SD	*n* or Mean	% or SD	*p*-Value
	461	%	326	%	
Gender					
Female	267	57.9%	175	53.7%	
Male	185	40.1%	143	43.9%	0.479
Missing	9	2.0%	8	2.5%	
BMI (*n* = 635)	18.8	3.41	18.9	3.61	0.572
Age	12.1	1.75	11.7	1.64	**0.003**
School					
School 1	201	43.6%	152	46.6%	0.401
School 2	260	56.4%	174	53.4%	
Missing	0	0.0%	0	0.0%	
Grade					
4	64	13.9%	59	18.1%	**0.007**
5	70	15.2%	52	16.0%	
6	80	17.4%	54	16.6%	
7	58	12.6%	55	16.9%	
8	77	16.7%	61	18.7%	
9	68	14.8%	33	10.1%	
10	44	9.5%	12	3.7%	
Missing	0	0.0%	0	0.0%	
Income (tögrögs)					
<500,000	118	25.6%	94	28.8%	0.560
510,000–1,000,000	238	51.6%	170	52.1%	
>1,000,000	83	18.0%	52	16.0%	
Missing	22	4.8%	10	3.1%	
Mother’s education					
Primary school	65	14.1%	64	19.6%	0.057
Secondary school, high school and vocational school	149	32.3%	112	34.4%	
College or more	168	36.4%	111	34.0%	
Missing	79	17.1%	39	12.0%	
Breakfast					
Every day	258	56.0%	204	62.6%	**0.036**
Sometimes	37	8.0%	14	4.3%	
Never	165	35.8%	109	33.4%	
Missing	1	0.2%	0	0.0%	
SDQ total					
Close to average	323	70.1%	211	64.7%	0.273
Slightly raised	48	10.4%	48	14.7%	
High	29	6.3%	23	7.1%	
Missing	61	13.2%	44	13.5%	
Exercise					
Every day	77	16.7%	48	14.7%	0.736
4–6 times a week	47	10.2%	37	11.3%	
2–3 times a week	118	25.6%	87	26.7%	
Once a week	77	16.7%	47	14.4%	
Never	114	24.7%	80	24.5%	
Missing	28	6.1%	27	8.3%	

SD: standard deviation; BMI: body mass index; SDQ: strengths and difficulties questionnaire. At the time of writing, 100,000 tögrögs ≈ 34 USD. *p*-values below 0.05 are shown in bold.

**Table 2:** Linear regression results for sociodemographic and dietary factors associated with academic performance in mathematics; multiple imputation applied (*n* = 787).

	Model 1		Model 2		Model 3	
	Coef	95% CI	Coef	95% CI	Coef	95% CI
Sweets						
Less than every day	ref	−	ref	−	ref	−
Every day	0.15	(0.01, 0.29)	0.12	(−0.02, 0.26)	0.15	(−0.02, 0.32)
Gender						
Female			ref	−	ref	−
Male			−0.34	(−0.48, −0.19)	−0.34	(−0.48, −0.2)
BMI			−0.02	(−0.04, 0.00)	−0.02	(−0.05, 0.00)
Income (tögrögs)						
≤500,000			ref	−	ref	−
510,000–1,000,000			0.11	(−0.06, 0.27)	0.11	(−0.06, 0.28)
>1,000,000			0.15	(−0.08, 0.37)	0.15	(−0.08, 0.39)
Mother’s education						
Primary school			ref	−	ref	−
Secondary school, high school and vocational school			−0.04	(−0.26, 0.18)	−0.03	(−0.28, 0.22)
College or more			0.08	(−0.11, 0.28)	0.07	(−0.13, 0.27)
Frequency of having breakfast						
Sometimes			0.14	(−0.14, 0.42)	0.15	(−0.14, 0.43)
Every day			0.16	(−0.13, 0.46)	0.18	(−0.12, 0.47)
Never			ref	−	ref	
SDQ total						
Close to average			ref	−	ref	−
Slightly raised			−0.23	(−0.44, −0.03)	−0.22	(−0.42, −0.01)
High			−0.34	(−0.62, −0.06)	−0.32	(−0.59, −0.04)
Exercise						
Every day			ref	−	ref	−
4–6 times a week			0.08	(−0.19, 0.34)	0.08	(−0.20, 0.35)
2–3 times a week			0.13	(−0.09, 0.35)	0.14	(−0.08, 0.36)
Once a week			0.11	(−0.14, 0.36)	0.08	(−0.17, 0.33)
Never			−0.08	−0.30, 0.13)	−0.08	−0.31, 0.14)
Instant noodle						
Less than every day					ref	−
Every day					0.01	−0.17, 0.19)
Vegetables						
Less than once a week					ref	−
More than once a week					−0.16	−0.30, −0.01)
Fruits						
3 or less times a week					ref	−
4 or more times a week					−0.06	(−0.21, 0.08)
Milk						
3 or less times a week					ref	−
4 or more times a week					−0.02	(−0.16, 0.13)
Potato chips						
Less than every day					ref	−
Every day					−0.08	(−0.22, 0.06)
Juice						
Less than once a week					ref	−
More than once a week					0.00	(−0.15, 0.14)
Mutton						
Less than every day					ref	−
Every day					0.00	(−0.14, 0.15)
Beef						
3 or less times a week					ref	−
4 or more times a week					0.00	(−0.15, 0.14)
Adjusted R-squared	0.01	(0.00, 0.02)	0.08	(0.04, 0.12)	0.06	(0.03, 0.10)

BMI: body mass index; Coef: coefficient; CI: confidence interval SD: standard deviation; SDQ: strengths and difficulties questionnaire. At the time of writing, 100,000 tögrögs ≈ 34 USD.

**Table 3:** Linear regression results for sociodemographic and dietary factors associated with academic performance in the Mongolian language; multiple imputation applied (*n* = 787).

	Model 1		Model 2		Model 3	
	Coef	95% CI	Coef	95% CI	Coef	95% CI
Sweets						
Less than every day	ref	−	ref	−	ref	−
Every day	0.25	(0.11, 0.39)	0.21	(0.08, 0.34)	0.25	(0.09, 0.41)
Gender						
Female			ref	−	ref	−
Male			−0.63	(−0.76, −0.49)	−0.63	(−0.76, −0.50)
BMI			−0.02	(−0.04, −0.01)	−0.02	(−0.05, 0.00)
Income						
≤500,000			ref	−	ref	−
510,000–1,000,000			0.17	(0.02, 0.33)	0.17	(0.01, 0.33)
>1,000,000			0.26	(0.05, 0.47)	0.26	(0.05, 0.48)
Mother’s education						
Primary school			ref	−	ref	−
Secondary school, high school and vocational school			0.04	(−0.17, 0.25)	0.04	(−0.19, 0.27)
College or more			0.09	(−0.10, 0.27)	0.08	(−0.11, 0.26)
Breakfast						
No			ref	−	ref	−
Every day			0.08	(−0.20, 0.35)	0.08	(−0.20, 0.35)
Sometimes			0.20	(−0.06, 0.47)	0.19	(−0.08, 0.46)
SDQ total						
Close to average			ref	−	ref	−
Slightly raised			−0.28	(−0.46, −0.09)	−0.26	(−0.45, −0.07)
High			−0.31	(−0.58, −0.05)	−0.3	(−0.56, −0.03)
Exercise						
Every day			ref	−	ref	−
4–6 times a week			0.13	(−0.12, 0.38)	0.14	(−0.12, 0.40)
2–3 times a week			0.19	(−0.01, 0.39)	0.20	(0.00, 0.40)
Once a week			0.20	(−0.03, 0.43)	0.17	(−0.07, 0.40)
Never			0.04	(−0.17, 0.24)	0.05	(−0.16, 0.25)
Instant noodle						
Less than every day					ref	−
Every day					−0.03	(−0.20, 0.13)
Vegetables						
Less than once a week					ref	–
More than once a week					−0.13	(−0.26, 0.01)
Fruits						
3 or less times a week					ref	−
4 or more times a week					−0.01	(−0.15, 0.12)
Milk						
3 or less times a week					ref	−
4 or more times a week					−0.01	(−0.14, 0.13)
Potato chips						
Less than every day					ref	−
Every day					−0.06	(−0.19, 0.07)
Juice						
Less than once a week					ref	−
More than once a week					−0.03	(−0.16, 0.11)
Mutton						
Less than every day					ref	−
Every day					−0.01	(−0.15, 0.13)
Beef						
3 or less times a week					ref	–
4 or more times a week					−0.06	(−0.19, 0.08)
Adjusted R-squared	0.02	(0.00, 0.04)	0.18	(0.13, 0.23)	0.18	(0.14, 0.24)

BMI: body mass index; Coef: coefficient; CI: confidence interval SD: standard deviation; SDQ: strengths and difficulties questionnaire. At the time of writing, 100,000 tögrögs ≈ 34 USD.

## Appendix A

**Table A1:** Pearson’s correlation coefficients between various foods and sweets consumed by students in the past month.

	rho
Sweets	1.00
Mutton	0.15
Beef	0.16
Ham sausage	0.17
Pork	0.16
Chicken	0.06
Milk	0.18
Yogurt	0.18
Aaruuleezgii (cheese)	0.10
Aarts (curds from sour milk)	0.12
Kumis (fermented dairy product)	0.09
Potato	0.16
Vegetables	0.09
Bread	0.11
Hiliinboov (solid bread)	0.24
Black bread	0.21
Instant noodle	0.42
Potato chips	0.21
Nuts	0.07
Berries	0.12
Fruits	0.11
Tea	0.18
Tea salt	0.11
Juice	0.20
Suuteitsai salt (milk tea with salt)	0.03

rho: Pearson’s correlation coefficient.
